# TIPE2 inhibits PDGF-BB-induced phenotype switching in airway smooth muscle cells through the PI3K/Akt signaling pathway

**DOI:** 10.1186/s12931-021-01826-5

**Published:** 2021-08-26

**Authors:** Huiyuan Wang, Bo Zhong, Yan Geng, Juanjuan Hao, Qiaoyan Jin, Yang Zhang, Lijuan Dong, Dan Gao, Jing Li, Wei Hou

**Affiliations:** Department of Pediatric, The Second Affiliated Hospital of Xi’an Jiaotong University, NO.157, Xiwu Road, Xi’an, 710004 Shaanxi China

**Keywords:** Childhood asthma, TIPE2, Airway smooth muscle cells (ASMCs), Phenotype switching, PI3K/Akt signaling pathway

## Abstract

**Background:**

Childhood asthma is a common respiratory disease characterized by airway inflammation. Tumor necrosis factor-α-induced protein 8-like 2 (TIPE2) has been found to be involved in the progression of asthma. This study aimed to explore the role of TIPE2 in the regulation of airway smooth muscle cells (ASMCs), which are one of the main effector cells in the development of asthma.

**Materials and methods:**

ASMCs were transfected with pcDNA3.0-TIPE2 or si-TIPE2 for 48 h and then treated with platelet-derived growth factor (PDGF)-BB. Cell proliferation of ASMCs was measured using the MTT assay. Cell migration of ASMCs was determined by a transwell assay. The mRNA expression levels of calponin and smooth muscle protein 22α (SM22α) were measured using qRT-PCR. The levels of TIPE2, calponin, SM22α, PI3K, p-PI3K, Akt, and p-Akt were detected by Western blotting.

**Results:**

Our results showed that PDGF-BB treatment significantly reduced TIPE2 expression at both the mRNA and protein levels in ASMCs. Overexpression of TIPE2 inhibited PDGF-BB-induced ASMC proliferation and migration. In addition, overexpression of TIPE2 increased the expression of calponin and SM22α in PDGF-BB-stimulated ASMCs. However, an opposite effect was observed with TIPE2 knockdown. Furthermore, TIPE2 overexpression blocked PDGF-BB-induced phosphorylation of PI3K and Akt, whereas the expression of p-PI3K and p-Akt were aggravated by TIPE2 knockdown. Additionally, the effects of TIPE2 overexpression and TIPE2 knockdown were altered by IGF-1 and LY294002 treatments, respectively.

**Conclusions:**

Our findings demonstrate that TIPE2 inhibits PDGF-BB-induced ASMC proliferation, migration, and phenotype switching via the PI3K/Akt signaling pathway. Thus, TIPE2 may be a potential therapeutic target for the treatment of asthma.

## Background

Childhood asthma is one of the most common chronic respiratory diseases occurring in children and is characterized by inflammation in the airways [1]. Classic asthma symptoms include cough, wheezing, and breathlessness in children, which significantly affect their quality of life [2]. Childhood asthma has been considered a burden globally, and its worldwide prevalence has increased in the last 2–3 decades [3].

The cause of asthma has been found to be complex with strong genetic and environmental components; however, its molecular mechanism is yet to be definitively characterized [4]. The key pathophysiological features of asthma are airway hyperresponsiveness and airway inflammation [5]. Recent data imply that airway smooth muscle (ASM) responses play an important role in asthma [6]. For example, ASM contraction leads to bronchoconstriction. The proliferation of airway smooth muscle cells (ASMCs) contributes to increased ASM mass during airway remodeling [7]. In addition, the pro-inflammatory mediators secreted by ASMCs perpetuate airway inflammation [8]. Taken together, ASMC phenotype plasticity can be considered a key hallmark of asthma pathogenesis. Preventing or reverting ASM modulation towards an asthma phenotype may be a novel therapeutic approach to control asthma.

Tumor necrosis factor-α-induced protein 8-like 2 (TIPE2) is a newly identified immune negative regulator that has been found to be involved in the progression of asthma. TIPE2 expression is significantly downregulated in peripheral blood mononuclear cells (PBMCs) of patients with bronchial asthma [9]. TIPE2 mRNA and protein expression is decreased in children with asthma and negatively correlated with immunoglobulin E, eosinophil (EO), and interleukin-4 levels, suggesting that reduced TIPE2 expression may contribute to the pathogenesis of childhood asthma [10]. However, the role of TIPE2 in regulating the phenotype switching of ASMCs remains unclear. This study attempted to provide an in-depth investigation of the role of TIPE2 in PDGF-BB-induced ASMC phenotype plasticity.

## Materials and methods

### Cell isolation, culture, and treatment

Primary ASMCs were isolated from the airways of four normal C57BL/6 mice. Briefly, the mouse trachea was isolated under sterile conditions. After washing with cold PBS, the trachea was digested with 0.2% collagenase IV (Sigma-Aldrich, St. Louis, USA) and 0.05% elastase (Sigma-Aldrich) for 30 min at 37 °C, followed by centrifugation. ASMCs were identified by the typical “hill and valley” growth pattern and immunocytochemical staining for α-smooth muscle actin. The pellet containing ASMCs was resuspended in Dulbecco’s Modified Eagle Medium (DMEM; Gibco, Rockville, MD, USA) containing 10% heat-inactivated fetal bovine serum (FBS; Gibco), 100 U/mL penicillin (Sigma-Aldrich), and 100 µg/mL streptomycin (Sigma-Aldrich) and was placed in a humidified 5% CO_2_ incubator for cell culture. The culture medium was changed every 3 days, and cells from passages 2–4 were used for all experiments. For the PDGF-BB treatment, cells were treated with various concentrations of PDGF-BB (0, 5, 10, 20, and 40 ng/mL) for 24 h.

### Plasmid construction and cell transfection

After digestion with 0.25% trypsin-EDTA solution, ASMCs were seeded into 6-well plates at a density of 5 × 10^4^/well and cultured for cell transfection until confluence reached 50–60%. TIPE2 open reading frame clones were cloned into the pcDNA3.0 vector to construct the TIPE2-overexpressing plasmid (pcDNA3.0-TIPE2). The constructed pcDNA3.0-TIPE2 plasmid was transfected into ASMCs to overexpress TIPE2. ASMCs transfected with the pcDNA3.0 plasmid were used as negative controls. TIPE2-knockdown ASMCs were constructed by transfection with specific siRNA-targeting TIPE2 (si-TIPE2) according to the RNA interference theory. ASMCs transfected with si-NC were used as negative controls. Lipofectamine™ 2000 (Life Technologies, Grand Island, NY, USA) was used for transient transfection.

### Quantitative RT-PCR (qRT-PCR)

Total RNA was isolated from ASMCs using the TRIzol Reagent (Ambion, Carsland, CA, USA). Complementary DNA (cDNA) was then synthesized using total RNA with a High-Capacity cDNA Reverse Transcription Kit (Applied Biosystems, Foster, CA, USA). qRT-PCR analyses were subsequently conducted to quantify the relative mRNA expression of TIPE2, calponin, and smooth muscle protein 22α (SM22α) using the SYBR Master Mix (Applied Biosystems) under the following conditions: 40 cycles of 95 °C for 30 s, 56 °C for 30 s, 65 °C for 1 min, and 72 °C for 5 min. β-Actin was used as an internal control for mRNA template normalization. The primer sequences for qRT-PCR were as follows: TIPE2, F, 5ʹ-GTGACTGACCACATACCCCA-3ʹ; R, 5ʹ-AGTGTTAGTGCCAGGTGAGC-3ʹ; calponin, F, 5ʹ-CGGGCACCAAGCGGCAGATCT-3ʹ; R, 5ʹ-CCGGGG TCAGGCAGTACTTGGGA-3ʹ; SM22α, F, 5ʹ-CCCGCCCTCCATGGTCTT CAAG-3ʹ; R, 5ʹ-GCCAAACTGCCCAAAGCCATTAC-3ʹ; and β-actin, F, 5ʹ-CATCACTATCGGCAATGAGC-3ʹ; R, 5ʹ-GACAGCACTGTGTTGGCATA-3ʹ. The relative expression of the target genes was determined using the 2^−ΔΔct^ method.

### Western blot

Cells were mixed with RIPA buffer (Yeasen Biotech, Shanghai, China) supplemented with protease and phosphatase inhibitors and then homogenized in an ice bath at 4 °C for 30 min. After centrifugation at 10,000×*g* at 4 °C for 20 min, the supernatant was obtained, and the protein concentration was measured using a bicinchoninic acid reagent kit (Yeasen Biotech). Equal amounts of protein (30 μg) were subjected to 10% SDS-PAGE and then transferred onto a nitrocellulose membrane. Standard Western blotting was conducted as previously described [11] using the primary antibodies anti-TIPE2 (1:1,500 dilution; Abcam, Cambridge, MA, USA), anti-calponin (1:2,500 dilution; Abcam), anti-SM22α (1:2,000 dilution; Abcam), anti-p-PI3K (1:1000 dilution; Cell Signaling Technology, Boston, MA, USA), anti-PI3K (1:1500 dilution; Cell Signaling Technology), anti-p-Akt (1:800 dilution; Sigma-Aldrich), anti-Akt (1: 2500 dilution; Sigma-Aldrich), anti-β-actin (1:1500 dilution; Cell Signaling Technology), and secondary antibody (1:1000 dilution; Cell Signaling Technology). Quantitative protein analysis was performed using ImageJ software (National Institutes of Health, Bethesda, MD, USA).

### Cell proliferation assay

ASMCs were digested with 0.25% trypsin-EDTA and seeded (5 × 10^3^ cells/well) in a 96-well plate in complete DMEM. Briefly, ASMCs were transfected with pcDNA3.0-TIPE2 or si-TIPE2 for 48 h and then treated with PDGF-BB. Subsequently, 5 mg/mL MTT (Sigma-Aldrich) was added to the cells for 4 h. The medium was replaced with 150 µL of DMSO and mixed thoroughly in the oscillator for 10 min to dissolve the formazan crystals. The absorbance at 490 nm wavelength was read using an ELISA reader (BioTek Instruments, Winooski, VT, USA).

### Cell migration assay

Transwell filter chambers with 8-µm pores (BD Biosciences, San Jose, CA, USA) were used for the migration assay. ASMCs (5 × 10^5^ cells/mL) in serum-free medium were seeded into the upper well of the chamber, and 500 μL of DMEM containing 10% FBS was added to the lower well. The chambers were then incubated at 37 °C for 24 h, and the cells were allowed to migrate. The outer side of the insert was gently rinsed with PBS, and the migrated cells were fixed with 4% paraformaldehyde (Santa Cruz Biotechnology, Santa Cruz, CA, USA) for 15 min at 25 °C and stained with 0.1% crystal violet solution (Sigma-Aldrich) for 15 min. Migrated cells were counted under a light microscope (Olympus, Tokyo, Japan) in five randomly chosen fields.

### Statistical analysis

All data were presented as mean ± SD. All statistical analyses were performed using the SPSS statistical analysis program (version 17.0; SPSS Inc., Chicago, IL, USA). Graphs were generated using GraphPad Prism software (version 5.0; GraphPad Software, San Diego, CA, USA). Differences between the two groups were compared using unpaired Student’s t-test. Differences among multiple groups were compared using a one-way analysis of variance. Statistical significance was set at p < 0.05.

## Results

### PDGF-BB treatment reduced TIPE2 expression in ASMCs

**Fig. 1:**
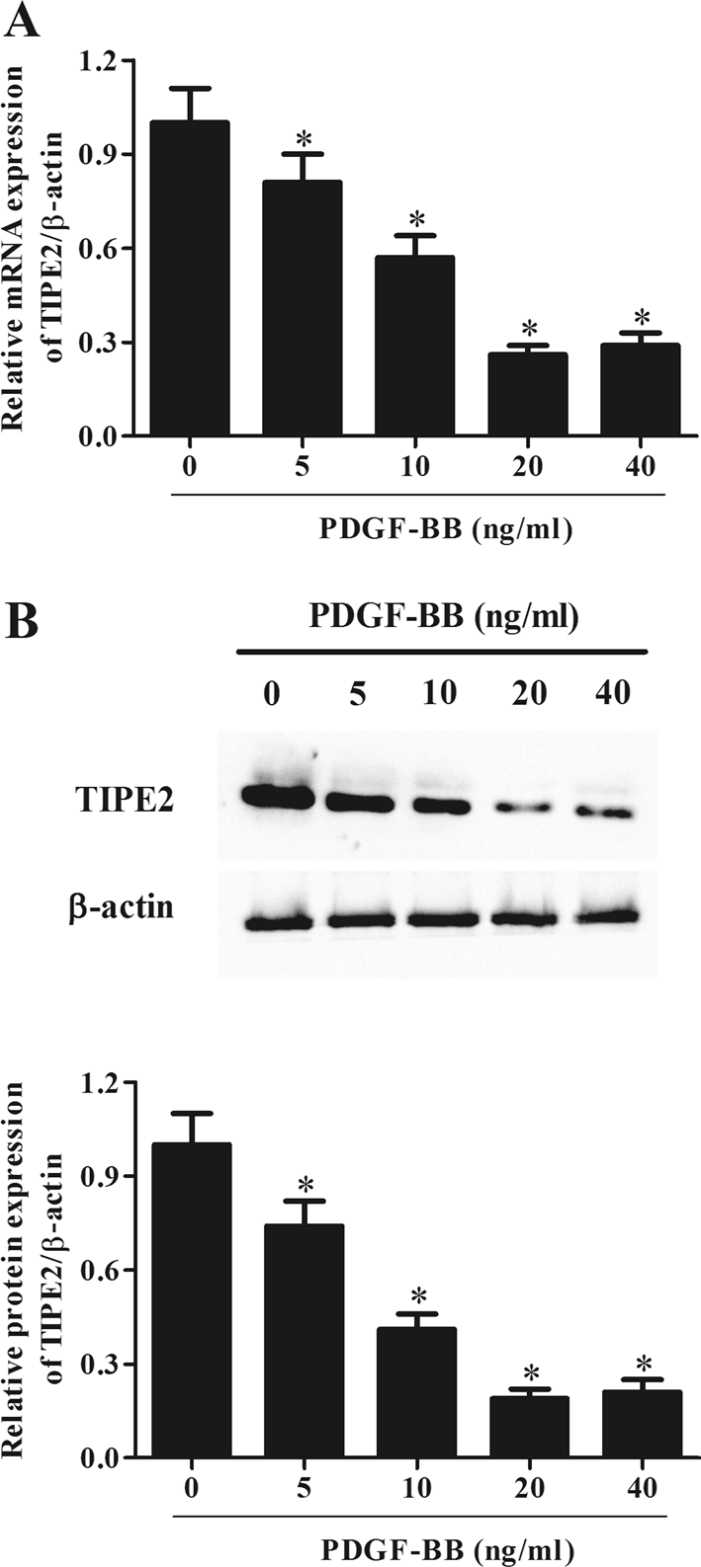
Tumor necrosis factor-α-induced protein 8-like 2 (TIPE2) expression in airway smooth muscle cells (ASMCs) with or without platelet-derived growth factor (PDGF)-BB treatment. ASMCs were treated with various concentrations of PDGF-BB (0, 5, 10, 20, and 40 ng/mL) for 24 h. The mRNA and protein expression levels of TIPE2 were detected through **A** qRT-PCR and **B** Western blotting, respectively. N = 4. *p < 0.05 in the control group

The effect of PDGF-BB on TIPE2 mRNA and protein expression was examined. ASMCs were treated with various concentrations of PDGF-BB (0, 5, 10, 20, and 40 ng/mL) for 24 h, and the expression levels of TIPE2 were detected using qRT-PCR and Western blotting, respectively. The results showed that PDGF-BB treatment significantly decreased the mRNA level of TIPE2 in a concentration-dependent manner (Fig. 1A). Similarly, the protein level of TIPE2 was also decreased in ASMCs after incubation with PDGF-BB (Fig. 1B). There was no significant difference between the 20 ng/mL PDGF-BB and 40 ng/mL PDGF-BB groups; thus, the 20 ng/mL PDGF-BB was selected for the following experiments.

### Overexpression of TIPE2 inhibited PDGF-BB-induced ASMC proliferation and migration

**Fig. 2:**
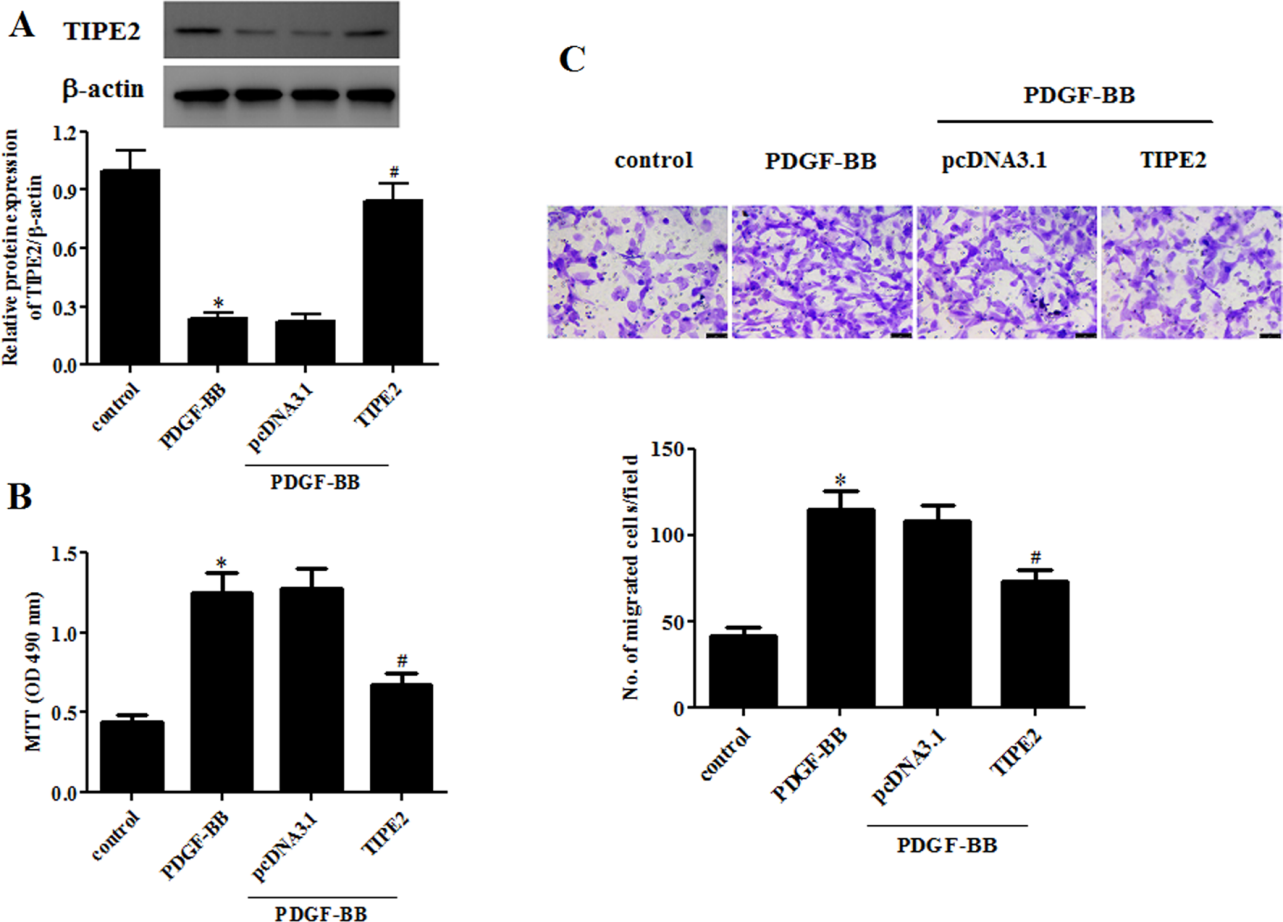
Overexpression of TIPE2 inhibited PDGF-BB-induced ASMC proliferation and migration. ASMCs were transfected with pcDNA3.0-TIPE2 or pcDNA3.0 for 48 h, and then they were treated with PDGF-BB. **A** The protein expression levels of TIPE2 were detected through Western blotting. **B** The cell proliferation of ASMCs was measured using the MTT assay. **C** Cell migration of ASMCs was determined using the transwell assay. Control group, *p < 0.05, vs. the pcDNA3.0 + PDGF-BB group, ^#^*p* < 0.05

We then examined the effects of TIPE2 overexpression on the proliferation and migration of ASMCs. TIPE2-overexpressing ASMCs were constructed by transfection with the TIPE2-overexpressing plasmid pcDNA3.0-TIPE2. As shown in Fig. 2A, pcDNA3.0-TIPE2 significantly increased the protein expression of TIPE2 in PDGF-BB-treated ASMCs. In addition, the results of the MTT assay indicated that cell proliferation of ASMCs was induced by PDGF-BB treatment, whereas cell proliferation was decreased in TIPE2-overexpressing ASMCs compared with that in PDGF-BB-induced ASMCs (Fig. 2B). In addition, the transwell migration assay demonstrated that TIPE2 overexpression greatly suppressed PDGF-BB-induced cell migration in ASMCs (Fig. 2C).

### Overexpression of TIPE2 elevated the PDGF-BB-mediated reduction of calponin and SM22α expressions in ASMCs

**Fig. 3:**
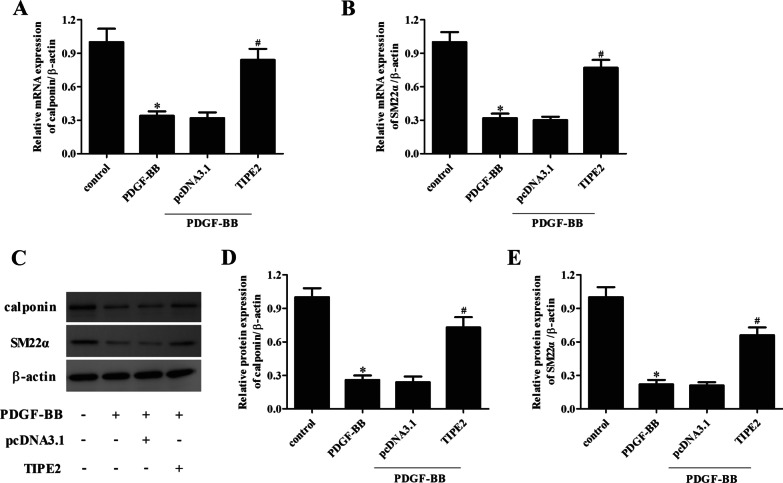
Overexpression of TIPE2 elevated the PDGF-BB-mediated reduction of calponin and SM22α expressions in ASMCs. ASMCs were transfected with pcDNA3.0-TIPE2 or pcDNA3.0 for 48 h and then treated with PDGF-BB. **A**, **B** The mRNA expression levels of calponin and SM22α were measured using the qRT-PCR assay. **C** The protein expression levels of calponin and SM22α were detected using the Western blot assay. **D**, **E** Quantification analysis of calponin and SM22α. Control group, *p < 0.05, vs. the pcDNA3.0 + PDGF-BB group, ^#^*p* < 0.05

Subsequently, we evaluated the effect of TIPE2 overexpression on the contractile phenotype switching of ASMCs. The expression levels of contractile phenotype marker proteins, including calponin and SM22α, were detected using qRT-PCR and Western blotting, respectively. The results in Fig. 3A, B show that the mRNA levels of calponin and SM22α in PDGF-BB-induced ASMCs were significantly lower than those in the control group. However, the decreased calponin and SM22α mRNA levels were elevated by TIPE2 overexpression. In line with the results of the qRT-PCR assay, the results of the Western blot assay indicated that TIPE2 overexpression markedly increased the protein expression levels of calponin and SM22α in PDGF-BB-treated ASMCs (Fig. 3C–E).

### Knockdown of TIPE2 enhanced PDGF-BB-induced ASMC proliferation and migration

**Fig. 4:**
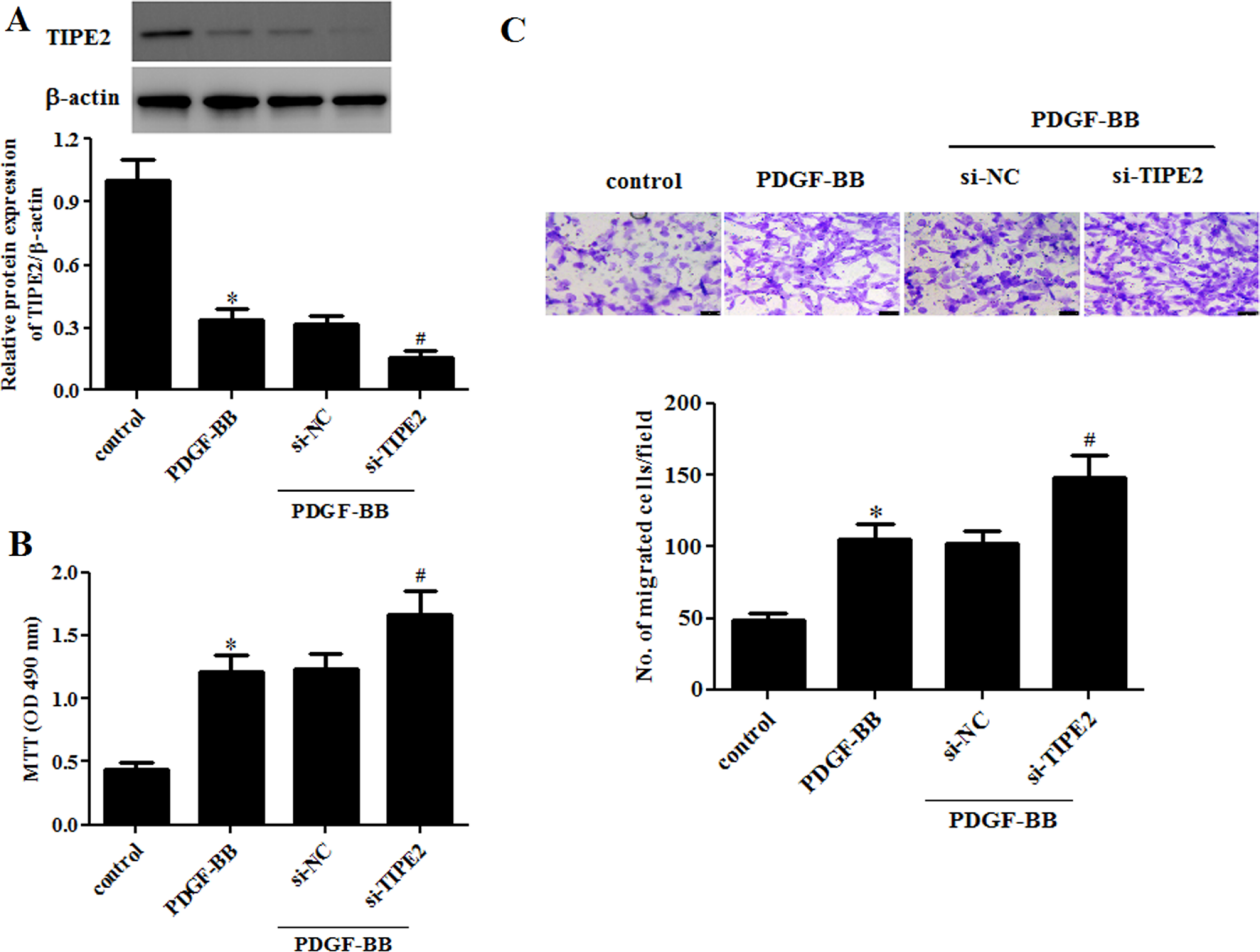
Knockdown of TIPE2 enhanced PDGF-BB-induced ASMC proliferation and migration. ASMCs were transfected with si-TIPE2 or si-NC for 48 h and then treated with PDGF-BB. **A** The protein expression levels of TIPE2 were detected through the Western blot assay. **B** Cell proliferation of ASMCs was measured using the MTT assay. **C** Cell migration of ASMCs was determined through the transwell assay. Control group, *p < 0.05, vs. the si-NC + PDGF-BB group, ^#^*p* < 0.05

To evaluate the role of TIPE2 knockdown in PDGF-BB-induced ASMCs, TIPE2-silencing ASMCs were established by transfection with si-TIPE2. Western blot results indicated that TIPE2 knockdown greatly decreased the protein expression level of TIPE2 in PDGF-BB-treated ASMCs (Fig. 4A). In addition, PDGF-BB significantly increased the proliferation of ASMCs, which was dramatically enhanced by TIPE2 knockdown (Fig. 4B). Moreover, TIPE2 knockdown aggravated the PDGF-BB-induced migration of ASMCs (Fig. 4C).

### Knockdown of TIPE2 aggravated PDGF-BB-mediated reduction in calponin and SM22α expressions in ASMCs

**Fig. 5:**
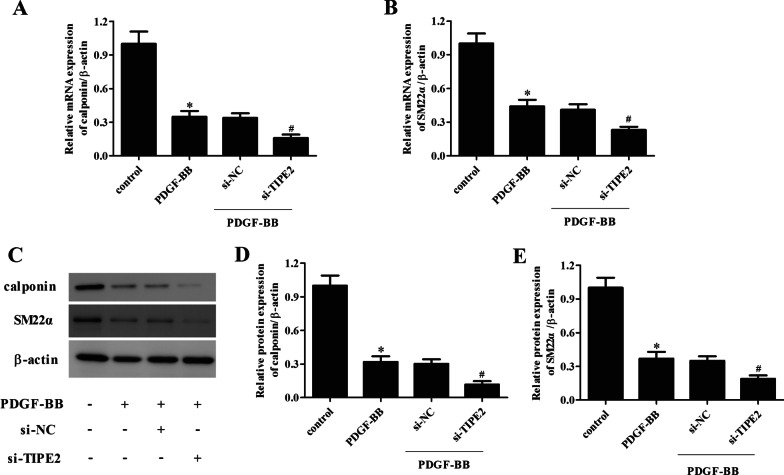
Knockdown of TIPE2 aggravated PDGF-BB-mediated reduction of calponin and SM22α expressions in ASMCs. ASMCs were transfected with si-TIPE2 or si-NC for 48 h and then treated with PDGF-BB. **A**, **B** The mRNA expression levels of calponin and SM22α were measured using the qRT-PCR assay. **C** The protein expression levels of calponin and SM22α were detected using the Western blot assay. **D**, **E** Quantification analysis of calponin and SM22α. Control group, *p < 0.05, vs. the si-NC + PDGF-BB group, ^#^*p* < 0.05

We then evaluated the effect of TIPE2 knockdown on the expression levels of calponin and SM22α in PDGF-BB-induced ASMCs. PDGF-BB treatment had significant inhibitory effects on the mRNA levels of calponin and SM22α in ASMCs. However, these inhibitory effects of PDGF-BB on calponin and SM22α mRNA expression levels were aggravated by TIPE2 knockdown (Fig. 5A, B). Similarly, knockdown of TIPE2 significantly aggravated the PDGF-BB-induced reduction in the protein expression levels of calponin and SM22α in ASMCs (Fig. 5C–E).

### Overexpression of TIPE2 inhibited PDGF-BB-induced activation of the PI3K/Akt signaling pathway in ASMCs

**Fig. 6:**
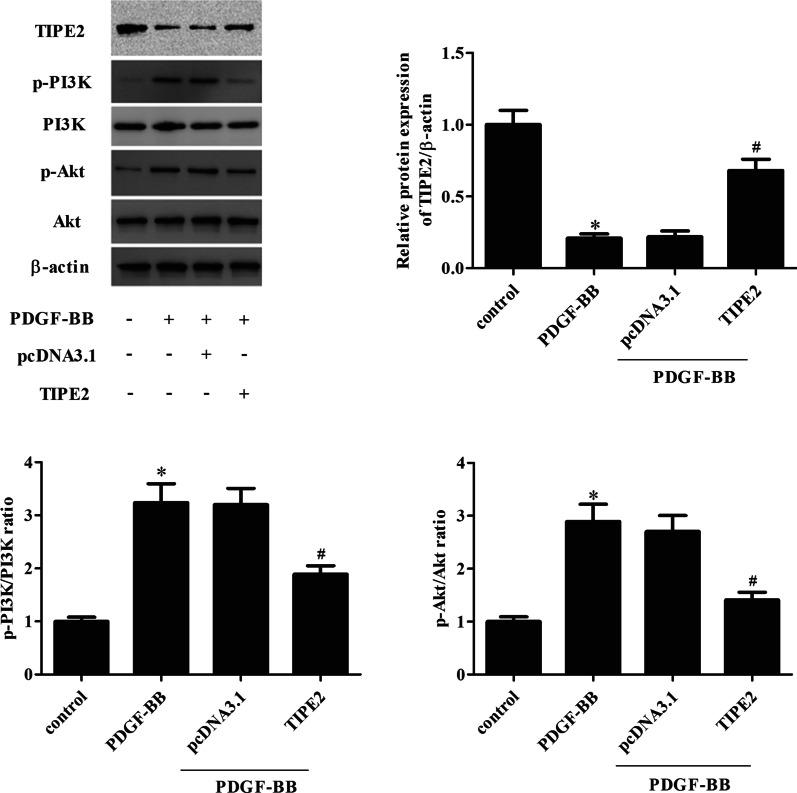
Overexpression of TIPE2 inhibited PDGF-BB-induced activation of the PI3K/Akt signaling pathway in ASMCs. ASMCs were transfected with pcDNA3.0-TIPE2 or pcDNA3.0 for 48 h and then treated with PDGF-BB. The levels of TIPE2, PI3K, p-PI3K, Akt, and p-Akt were determined through Western blotting. Control group, *p < 0.05, vs. the pcDNA3.0 + PDGF-BB group, ^#^*p* < 0.05

The PI3K/Akt signaling pathway has been demonstrated to be associated with ASMC phenotype switching [12, 13]. Therefore, we investigated whether TIPE2 could influence the PI3K/Akt signaling pathway in ASMCs. As indicated in Fig. 6, PDGF-BB treatment greatly increased the levels of p-PI3K and p-Akt in ASMCs, which were significantly reduced after transfection with pcDNA3.0-TIPE2.

### IGF-1 treatment reversed the effect of TIPE2 on ASMC phenotype switching

**Fig. 7:**
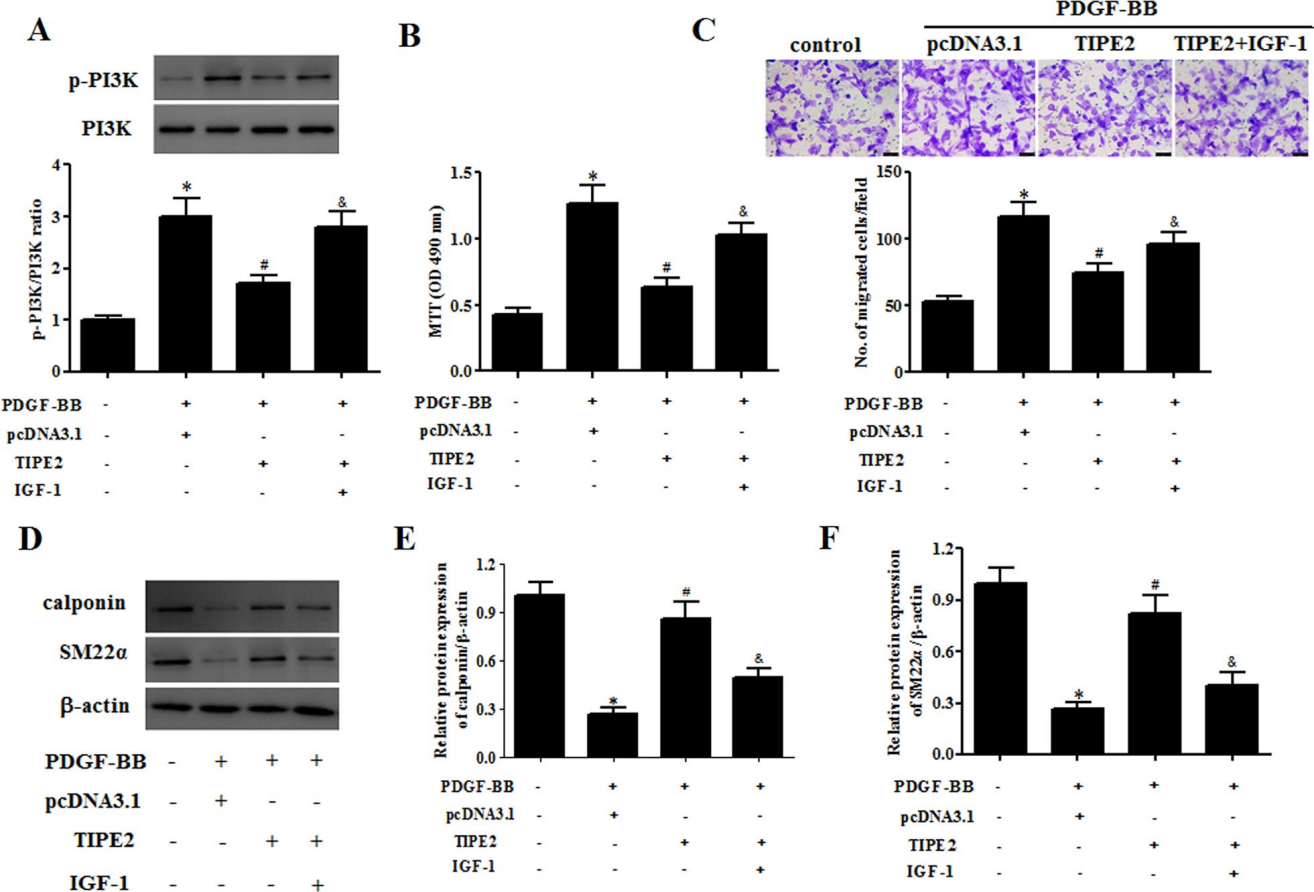
IGF-1 treatment reversed the effect of TIPE2 on ASMC phenotype switching. ASMCs were transfected with pcDNA3.0-TIPE2 for 48 h in the presence of IGF-1 (5 μM) and then treated with PDGF-BB. **A** The expression levels of PI3K and p-PI3K in ASMCs were determined by Western blotting. **B** The cell proliferation of ASMCs was measured by the MTT assay. **C** Cell migration of ASMCs was determined using the transwell assay. **D** The protein expression levels of calponin and SM22α in ASMCs were determined by Western blotting. **E**, **F** Quantification analysis of calponin and SM22α. Control group, *p < 0.05, vs. the pcDNA3.0 + PDGF-BB group, ^#^*p* < 0.05, vs. the pcDNA3.0-TIPE2 + PDGF-BB group, ^&^*p* < 0.05

The ASMCs were then treated with IGF-1 to mediate the activation of the PI3K/Akt signaling pathway. We observed that IGF-1 increased the level of p-PI3K inhibited by TIPE2 overexpression in PDGF-BB-treated ASMCs (Fig. 7A). In addition, MTT and transwell assay results indicated that the decreased cell proliferation and migration in TIPE2-overexpressing ASMCs were reversed by IGF-1 in PDGF-BB-treated ASMCs (Fig. 7B, C). Furthermore, an increase in the protein levels of calponin and SM22α caused by TIPE2 overexpression was attenuated by IGF-1 in PDGF-BB-treated ASMCs (Fig. 7D–F).

### Knockdown of TIPE2 aggravated PDGF-BB-induced activation of PI3K/Akt signaling pathway in ASMCs

**Fig. 8:**
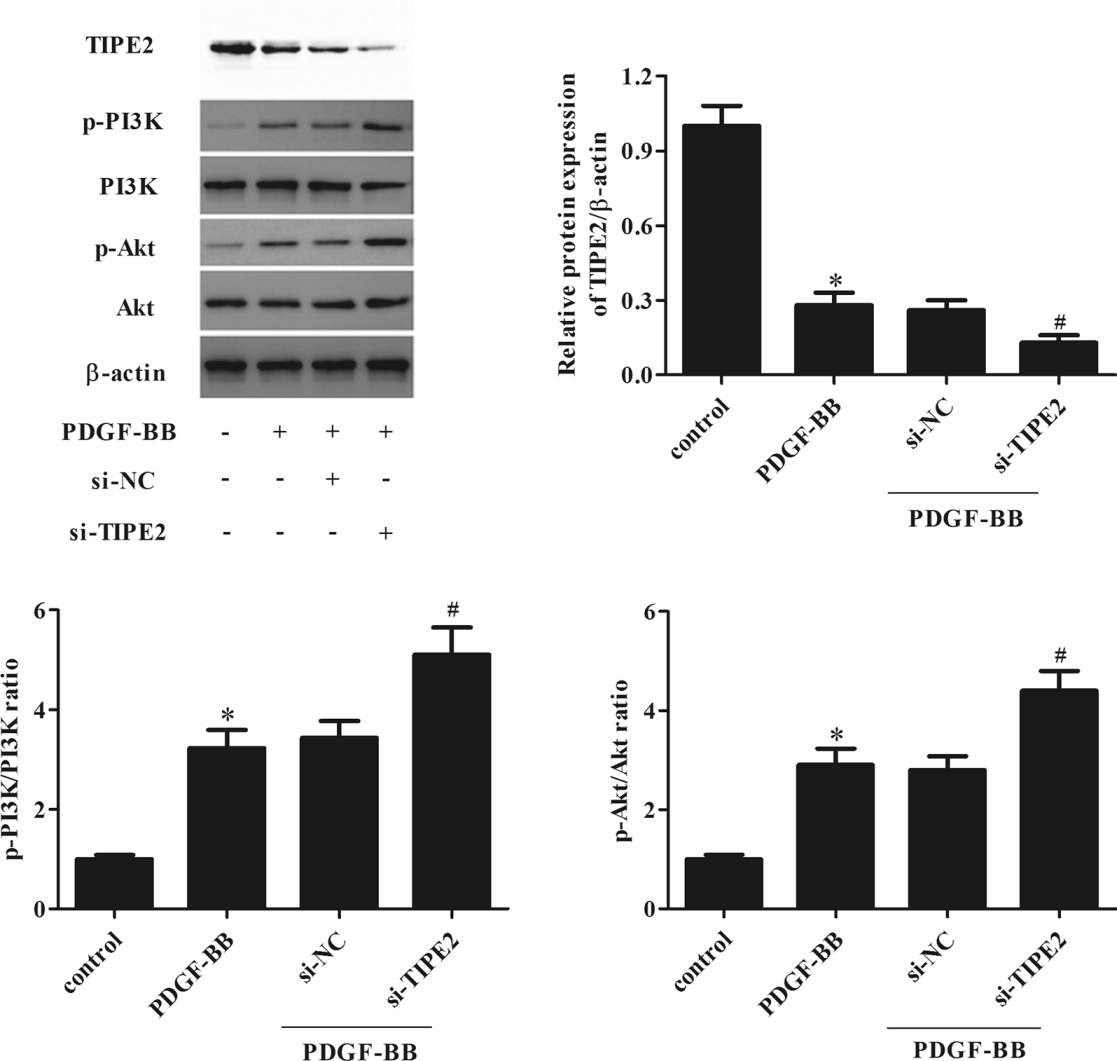
Knockdown of TIPE2 aggravated PDGF-BB-induced activation of PI3K/Akt signaling pathway in ASMCs. ASMCs were transfected with si-TIPE2 or si-NC for 48 h and then treated with PDGF-BB. The levels of TIPE2, PI3K, p-PI3K, Akt, and p-Akt were determined by Western blotting. Control group, *p < 0.05, vs. the si-NC + PDGF-BB group, ^#^*p* < 0.05

Additionally, the effect of TIPE2 knockdown on the PI3K/Akt signaling pathway was examined by detecting the levels of PI3K, p-PI3K, Akt, and p-Akt. Western blot results showed that the PDGF-BB-induced expression levels of p-PI3K and p-Akt were significantly enhanced by TIPE2 knockdown in ASMCs (Fig. 8).

### LY294002 reversed the effect of TIPE2 knockdown on the ASMC phenotype switching

**Fig. 9:**
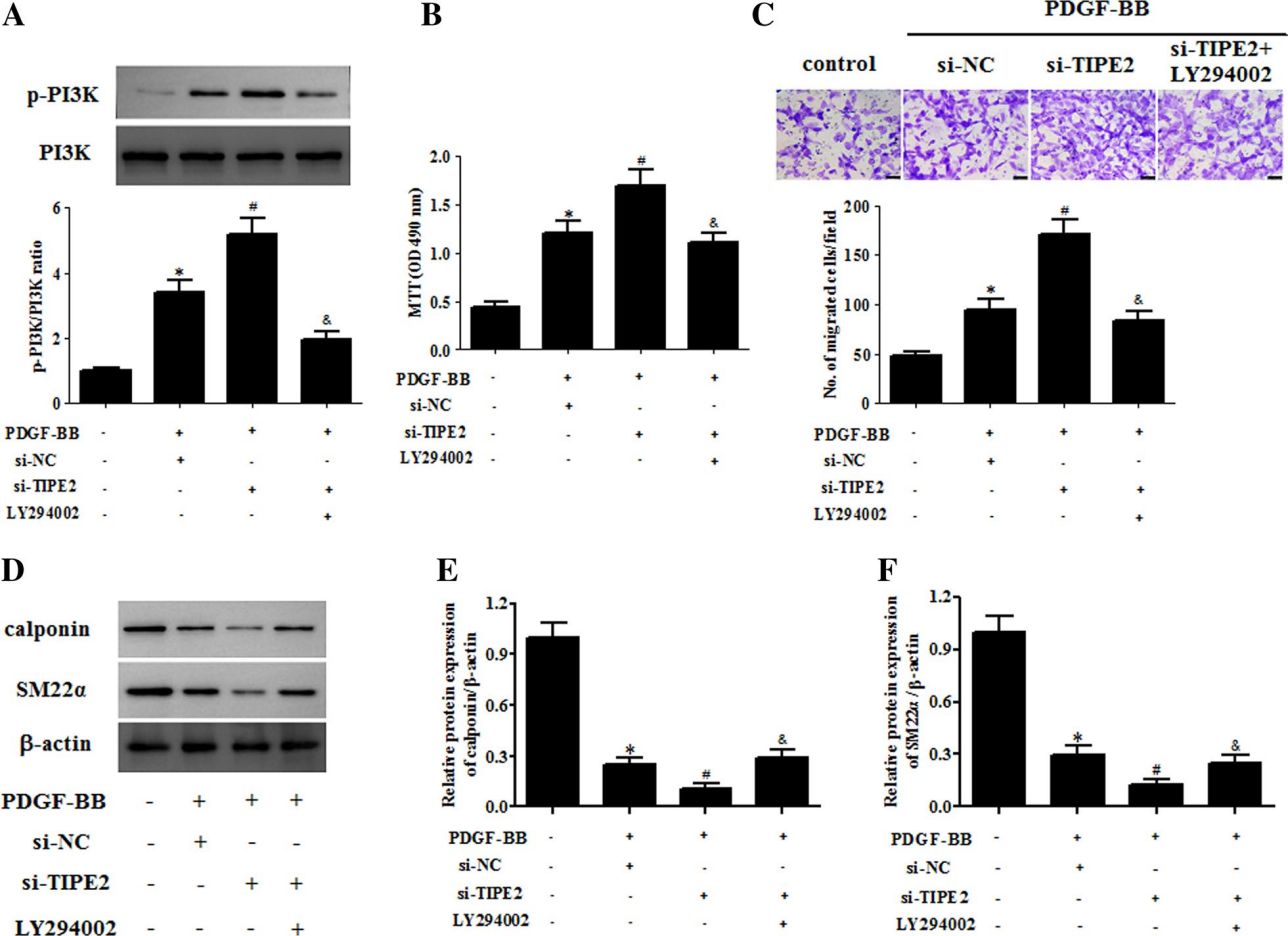
LY294002 reversed the effect of TIPE2 knockdown on ASMC phenotype switching. ASMCs were transfected with si-TIPE2 for 48 h in the presence of LY294002 (10 μM) and then treated with PDGF-BB. **A** The expression levels of PI3K and p-PI3K in ASMCs were determined by Western blotting. **B** Cell proliferation of ASMCs was measured by the MTT assay. **C** Cell migration of ASMCs was determined using the transwell assay. **D** The protein expression levels of calponin and SM22α in ASMCs were determined by Western blotting. **E**, **F** Quantification analysis of calponin and SM22α. Control group, *p < 0.05, vs. the si-NC + PDGF-BB group, ^#^*p* < 0.05, vs. the si-TIPE2 + PDGF-BB group, ^&^*p* < 0.05

The ASMCs were treated with a PI3K/Akt signaling pathway inhibitor, LY294002, to block its activation. As shown in Fig. 9A, the promotive effect of TIPE2 knockdown on the level of p-PI3K was inhibited by LY294002 in PDGF-BB-treated ASMCs (Fig. 9A). In addition, the increased cell proliferation and migration of ASMCs caused by si-TIPE2 was mitigated by LY294002 (Fig. 9B, C). Furthermore, reduction in the expression levels of calponin and SM22α due to TIPE2 knockdown was attenuated by LY294002 (Fig. 9D–E).

## Discussion

Allergic asthma is a chronic inflammatory disease characterized by airway hyperresponsiveness, EO infiltration, mucus hypersecretion, reversible airflow obstruction, airway remodeling, and goblet cell hyperplasia [14]. PDGF-BB is secreted by activated platelets as well as by endothelial, epithelial, glial, or inflammatory cells and is a major stimulus for pathologic cell proliferation and tissue remodeling [15–17]. ASMCs are one of the main effector cells in airway remodeling during asthma [18, 19]. PDGF-BB has been reported to be significantly upregulated in asthmatic tissues and initiates a multitude of biological effects that contribute to ASMC proliferation and migration, resulting in the progression of asthma [20–22]. Hence, blocking PDGF-BB-induced changes in ASMCs may prevent the development of asthma. In addition, recombinant human PDGF-BB was introduced in the experiments as a stimulator of asthma [23]. In the current study, PDGF-BB was used to induce ASMC proliferation, migration, and phenotype switching. Our results also proved that PDGF-BB treatment caused a significant reduction in TIPE2 expression. Overexpression of TIPE2 inhibited PDGF-BB-induced ASMC proliferation and migration, as well as reversed the PDGF-BB-suppressed expression of calponin and SM22α.

Several studies have shown that many molecular signaling pathways, such as JAK/STAT/MAPK, NF-κB, Wnt/β-catenin, PI3K/Akt, JNK, Fas/FasL, TLRs/MyD88, and Keap1/Nrf2/ARE, are important in the pathophysiology of asthma [14, 24–28]. Targeted therapy modulating cell signaling pathways can be a powerful strategy in designing new drugs to treat asthma. TIPE2 has been reported to be a negative regulator of the PI3K/Akt signaling pathway. TIPE2 inhibits gastric cancer by regulating cell proliferation, apoptosis, and inflammation through inhibition of the PI3K/Akt and Ras-Raf-MEK-ERK1/2 signaling pathways [29]. TIPE2 suppresses the proliferation, migration, and invasion of prostate cancer cells by inhibiting the PI3K/Akt signaling pathway [30]. TIPE2 controls innate immunity to RNA by targeting the PI3K signaling pathway [31]. TIPE2 suppresses atherosclerosis by exerting a protective effect on oxidized low-density lipoprotein-induced macrophages through the inhibition of the PI3K/Akt and NF-κB signaling pathways [32]. Therefore, we hypothesized that the PI3K/Akt signaling pathway might be associated with TIPE2-mediated regulation of ASMC phenotype switching. Our results proved that TIPE2 overexpression inhibits PDGF-BB-induced activation of the PI3K/Akt signaling pathway, whereas knockdown of TIPE2 aggravated activation of the PI3K/Akt signaling pathway in PDGF-BB-induced ASMCs. Moreover, IGF-1 treatment reversed the effect of TIPE2 overexpression on ASMC phenotype switching; however, LY294002 reversed the effect of TIPE2 knockdown on ASMC phenotype switching. Taken together, the effects of TIPE2 overexpression on ASMC phenotype switching were mediated by the PI3K/Akt signaling pathway.

## Conclusion

These results demonstrated that TIPE2 inhibited PDGF-BB-induced ASMC proliferation, migration, and phenotype switching by inhibiting the PI3K/Akt signaling pathway.

## Data Availability

The data sets used and/or analyzed during the current study are available from the corresponding author on reasonable request.
